# Medicinal Properties and Bioactive Compounds from Wild Mushrooms Native to North America

**DOI:** 10.3390/molecules26020251

**Published:** 2021-01-06

**Authors:** Mehreen Zeb, Chow H. Lee

**Affiliations:** Chemistry and Biochemistry Program, University of Northern British Columbia, Prince George, BC V2N 4Z9, Canada; zeb@unbc.ca

**Keywords:** wild mushrooms, fungi, medicinal mushrooms, bioactive compounds, North America

## Abstract

Mushrooms, the fruiting bodies of fungi, are known for a long time in different cultures around the world to possess medicinal properties and are used to treat various human diseases. Mushrooms that are parts of traditional medicine in Asia had been extensively studied and this has led to identification of their bioactive ingredients. North America, while home to one of the world’s largest and diverse ecological systems, has not subjected its natural resources especially its diverse array of mushroom species for bioprospecting purposes: Are mushrooms native to North America a good source for drug discovery? In this review, we compile all the published studies up to September 2020 on the bioprospecting of North American mushrooms. Out of the 79 species that have been investigated for medicinal properties, 48 species (60%) have bioactivities that have not been previously reported. For a mere 16 selected species, 17 new bioactive compounds (10 small molecules, six polysaccharides and one protein) have already been isolated. The results from our literature search suggest that mushrooms native to North America are indeed a good source for drug discovery.

## 1. Introduction

Natural products have been used as medicines for centuries, however, only in the last century have researchers begun to diligently characterize their biological and chemical properties. Mushrooms are natural reservoirs of potent pharmaceuticals and are now the new interface for drug discovery. Mushrooms belong to ascomycetes and basidiomycetes phyla of the kingdom Fungi, and have been defined as “*epigeous and hypogeous fruiting bodies of macroscopic fungi*” [1]. According to the recent estimates, fungi constitute 2.2–3.8 million species worldwide [2]. These fungal estimates include all the different types of fungi including mushrooms, the fruiting bodies of fungi. Another study provides estimates on the total number of mushrooms to be 140,000–160,000 where only 10% have been explored [3,4].

Mushrooms have a long history of medicinal use in various cultures across the globe. It has earned medicinal status long ago in China and other parts of Asia, including Japan and Korea. The scope of medicinal mushrooms has expanded to other countries such as USA and the eastern European countries such as Russia [5,6,7,8,9]. Initially, mushrooms became popular as a folklore remedy. For example, in the sixteenth century in Russia and Europe, *Inonotus obliquus* became famous as a folklore medicine for the treatment of cancer [10]. Later on, scientists became interested in finding the evidence behind the diverse bioactive potential that is contained in mushrooms. This has led to the exploration of untapped mushroom resources for their potential medicinal benefit and the bioactive compounds that impart these properties. There are many excellent recent reviews written on the topic of medicinal mushrooms and the bioactive compounds that derived from them [11,12,13,14]. Unlike in Asia and parts of Eastern Europe, there are relatively less studies on the exploration of mushrooms native to North America for medicinal properties.

The purpose of this comprehensive review is to provide an up-to-date information on the bioprospecting efforts on mushrooms native to North America. With literature searches and the information gained, we aim to critically answer the following questions: (i) Do similar species found in North America and elsewhere exhibit similar bioactivities and produce similar group of bioactive compounds?, (ii) Do similar species found in North America and elsewhere exhibit distinct bioactivity and produce distinct compounds?, (iii) Do new species found in North America produce new compound(s), and (iv) Based on the answers to (i) to (iii) above, is it worth exploring mushrooms native to North America for medicinal properties and for new medicinal compounds?

To review and answer the above questions, we performed literature search on the following database; Google Scholar, Science Direct, and PubMed. Some of the key search terms used included “medicinal mushrooms from North America”, “North American mushrooms”, “bioactive compounds from North American mushrooms”, “small molecules from mushrooms”, “large molecular weight compounds from medicinal mushrooms”, “medicinal mushrooms of British Columbia”, “medicinal mushrooms of Canada”, “medicinal mushrooms and United States”, and “medicinal mushrooms and Greenland”. The search was limited to the period from 2005–2020 and articles that have irrelevant topic such as morphology, taxonomy and ecological aspects of mushrooms were excluded. Research articles that involved biological activity and chemical characterization were included.

## 2. Mushrooms Native to North America

North America covering the northern subcontinent of the Americas that includes Canada, the United States, and Greenland, has one of the world’s largest and diverse ecological system. It is also home to diverse mushrooms that are relatively unexplored for their therapeutic benefit. Although new species continue to be discovered in North America, about 22–55% of the mushroom species remain unexplored [3]. Like elsewhere, mushrooms were recognized as an important source for medicine by people who lived in North America centuries ago. For example, the indigenous people of North America had used *Calvatia* mushrooms (more commonly known as puffballs) to heal wounds [15]. The therapeutic value of *Fomitopsis officinalis* was also discovered by First Nations peoples of North America, including those in British Columbia (BC) where *F. officinalis* sporophores were carved as shaman grave guardians [16].

In the recent years, there has been increasing reports on mushrooms native to North America possessing medicinal properties. Whether it be *Hericium* sp. which is found growing on hardwood and coniferous trees that contains a number of small molecules with antibacterial properties [17] or *Echinodontium tinctorium* which is native to BC commonly found growing as a woody conk on true firs with anti-inflammatory compound in its fruiting bodies [18]. Others include *Cortinarius armillatus* which grows in moist coniferous forests and contains orellanine, a potential toxin against renal carcinoma [19,20]. In Table 1, we summarize all the mushrooms found in North America that have been reported to possess medicinal properties, their origin of collection, types of bioactive components, and their active doses.

## 3. Medicinal Properties of Mushrooms Native to North America

Mushrooms are known to possess medicinal properties that provide benefits against a large number of diseases. Some of the important medicinal benefits reported include antimicrobial, antioxidant, anticancer, immune system enhancer, antiviral, anti-hyperlipidemia, radical scavenger, anti-parasitic and anti-inflammatory [42]. Amongst these, the most common medicinal properties reported from mushrooms native to North America are anti-cancer, immuno-stimulatory, anti-inflammatory, antimicrobial and antioxidant as shown in Table 1 [17,18,19,20,21,22,23,24,25,26,27,28,29,30,31,32,33,34,35,36,37,38,39,41].

### 3.1. Anti-Bacterial and Anti-Viral Activities

Antimicrobial resistance is a major healthcare problem worldwide. A recent landmark report indicated that bacterial infections resistant to treatment are likely to grow from 26% in 2018 to 40% By 2050, and such increases are expected to cost thousands of lives, billions of dollars in hospital expenses and gross domestic product and have a negative social impact on people worldwide [43]. Therefore, it is recommended that efforts to discover new antimicrobial drugs to combat specific antibiotic-resistant pathogens should be strengthened and should include innovative strategies [43,44,45]. Many studies around the world had focused on isolating antibacterial compounds and extracts from basidiomycete mushrooms [46]. For instance, the small molecules enokipodins A–D, isolated from *Flammulina velutipes* had activity against *Bacillus subtilis* and *Staphylococcus aureus* [47], while others including emodin, physcion, and erythroglaucin, austrocortilutein, torosachrysone, and 6-methylxanthopurpurin-3-*O*-methyl ether isolated from *Cortinarius* species were effective against *S. aureus* [48]. Small molecules from *Lentinus edodes* (Shiitake mushroom) have also shown antibacterial effects against *S. aureus, Streptococcus faecalis* and *Bacillus cereus* [49].

Not surprisingly, researchers have also focused on mushrooms native to North America, as avenues to discover novel antimicrobial metabolites. Grifolin, neogrifolin and confluentin isolated from *Albatrellus flettii* collected in California were found to have strong activity against gram positive bacteria *B. cereus* and *Enterococcus faecalis* [21]. Another lanostane-type tripterpene isolated from *Jahnoporus hirtus* also inhibited the growth of *B. cereus* and *E. faecalis* [21]. Other anti-bacterial small molecules isolated include lanostane triterpenoids and ergostane steroid from *Fomitopsis pinicola* collected in Oregon [30], new subclass of bisnaphthalenes from *Urnula craterium* [41] collected in La Crosse (WI, USA) and 2-aminoquinoline from *Leucopaxillus albissimus* [36]. 2-aminoquinoline showed modest antibacterial activity against multidrug resistant clinical isolates with an MIC of 8–64 μg/mL and weak antibacterial activity against *A. baumannii*, using tetracycline as a reference antibacterial agent [36]. The antibacterial activity of 2-aminoquinoline was not very convincing due to its high MIC, therefore, it was not suggested for further drug screening as an antibacterial agent in its current form. The antibacterial activity of small molecules from *F. pinicola* was compared with tetracycline [30]. Vancomycin and tetracycline were used as positive controls to compare the antimicrobial activity of urnucratin A-C isolated from *Urnula craterium* [41]. Amongst these compounds, urnucratin A (26) was the most promising with MIC values of 2, 1, and 0.5 μg/mL against *S. aureus*, *E. faecium* and *S. pyogenes* respectively [41]. It had an IC50 value greater than 100 μM against J774A.1 murine monocyte cells, indicating that it lacks non-specific toxicity [41]. Supernatants from culture of *Lenzites betulina* and *Haploporus odorus* [33] as well as extracts from *Pleurotus ostreatus* and *P. levis* [39] also have antimicrobial activity. However, the responsible antimicrobial compounds have not been isolated from these mushrooms. In another study using extracts from 75 mushrooms collected in Oxford (OH, USA), it was found that a total of 25 samples had antibacterial activity against at least one of the bacterial strains assessed [26]. From this study, extracts from *Ganoderma lucidum* and *Laetiporus sulphureus* were found to have the strongest antibacterial activity [26]. MIC was determined for these mushroom extracts using kanamycin as a reference antibacterial agent which has an MIC of 0.01 mg/mL against *S. epidermidis* [26]. Four small molecules Ramariolides A–D (22–25) were isolated from methanol extracts of *Ramaria cystidiophora* [50]. The major metabolite ramariolide A (21) was found to be active against *Mycobacterium smegmatis* and *Mycobacterium tuberculosis* with MIC of 8 μg/mL and 64−128 μg/mL respectively. This compound appears to be promising because isoniazid, a first-line antituberculosis bactericidal antibiotic also has an MIC of 8 μg/mL against *M. smegmatis* [50]. The authors did not report any antibacterial from ramariolides B–D (22–24), and therefore it is currently unknown whether these compounds have any biological activity.

Extracts of polypore mushrooms native to North America have been shown to have antiviral activities against viruses that attack honeybees [51]. Mycelium extracts from *Fomes fomentarius* collected in Ithaca (NY, USA) and *Ganoderma resinaceum* culture from Ontario (Canada) were found to reduce the levels of honeybee deformed wing virus and Lake Sinai virus in vivo in both laboratory and field studies [51].

### 3.2. Anti-Proliferative Activity

#### 3.2.1. Anti-Proliferative Activity In-Vitro

Mushrooms native to North America have been explored for anti-proliferative activity against cancer cell lines. For instance, out of 29 species of mushrooms examined from north-central BC [24] and Haida Gwaii, BC [23], 27 species exhibited anti-proliferative activity. Sixteen out of the 27 species (59%) had their anti-proliferative activity reported for the first time [23,24]. These species include *Amanita augusta, Cantharellus cibarius, Chroogomphus tomentosus, Guepinia helvelloides, Gyromitra esculenta, Hydnellum* sp., *Inocybe* sp., *Laetiporus conifericola, Leucocybe connata, Phellodon atratus, Pleurotus ostreatus, Ramaria cystidiophora, Russula paludosa, Trichaptum abietinum, Tricholomopsis rutilans*, and *Tyromyces chioneus*. It would be of interest to further investigate what the anti-proliferative compounds from these mushrooms are. Another study was conducted on 38 species of mushrooms collected from the greater Seattle area (WA, USA). Out of 38 species, aqueous extracts from three species were identified as anti-proliferative in human estrogen receptor negative (MDA-MB-231, BT-20) and estrogen receptor positive (MCF-7) breast cancer cells. These included *Coprinus comatus*, *Coprinellus* sp., and *Flammulina velutipes* [27]. The anti-proliferative effects of these 3 species were compared with 5-fluorouracil, a known anticancer drug [27]. Elsewhere, the ethanol and water extracts of *Pleurotus tuber-regium* from Washington State displayed anti-proliferative effects in HCT-116 colon and HeLa cervical cancer cell lines [52]. Although in most parts the identity of anti-proliferative compounds from the mushroom species described above remains unknown, there were detailed structural elucidation and mechanistic studies of both bioactive small molecules and polysaccharides isolated from selected species. For example, a growth-inhibitory polysaccharide GIPinv that caused growth inhibition in several cancer cell lines and induced apoptosis in HeLa cancer cells was isolated from *Paxillus involutus* [37]. It has an IC50 of 0.05 mg/mL and 0.04 mg/mL against HeLa cells and MCF-7 cells respectively. In the future, GIPinv should be evaluated for its anticancer activity in animal models. Small molecules grifolin, neogrifolin and confluentin were found to be the major growth-inhibitory compounds in the ethanol extracts of *Albatrellus flettii* [22]. It was also discovered that confluentin can inhibit the RNA-binding function of the oncogenic protein insulin-like growth factor 2 mRNA-binding protein 1 (IMP1) [22]. This is potentially a novel inhibitory pathway leading to the suppression of KRAS expression in human colon cancer cells. To determine the significance of this finding, it will be important to evaluate the inhibitory function of confluentin using in vivo models. Elsewhere, an exopolysaccharide isolated from *P. tuber-regium* inhibited the growth of chronic myelogenous leukemia K562 cells [40]. Another example is orenalline, a small molecule isolated from *Cortinarius armillatus* that inhibited renal carcinoma in a dose-dependent manner [20].

#### 3.2.2. Anti-Proliferative/Anti-Cancer Activity in Animal Models

To the best of our knowledge, to date there has been no animal studies for anticancer activity of any compounds isolated from North American mushroom. There are numerous animal studies reporting the anti-cancer activity of extracts, small molecules and large molecules isolated from mushrooms collected in different parts of the world. Several excellent reviews have already dealt with this topic [11,12,13,14]. Here, we only highlight some of the key findings from popular medicinal mushrooms. A mushroom mixture named as Micotherapy U-care consisted of *G. lucidum, Lentinula edodes, Agaricus blazei, Ophiocordyceps sinensis,* and *Grifola frondosa* was orally administered in mice implanted with triple-negative breast cancer. The results showed reduced pulmonary metastases density as well as decreased expression of interleukin-6, NOS, and COX-2 [53]. In another study, the anticancer effect of β-glucans along with radiation therapy was evaluated against lung cancer. The β-glucan isolated from *G. lucidum* caused inhibition of primary lung cancer metastasis in C57BL/6 mice [54]. In another study, the antitumor effects of a β-glucan isolated from *G. frondosa* combined with cisplatin were evaluated in a BALB/cA mouse model. The β-glucan was able to enhance the antitumor and antimetastatic effects of cisplatin as well as reduced the side effects; myelosuppression and nephrotoxicity, associated with cisplatin treatment [55]. The ethanol extracts from *F. pinicola* has been shown to significantly inhibit xenograft sarcoma-derived tumor growth in mice and prolong their survival time when administered as a food supplement [56].

*Hericium erinaceus* is one of the medicinal mushrooms that has been most extensively studied in animal models [11]. For example, its ethanol extract was able to inhibit gastric, liver and colon cancer in mouse xenograft tumors [57] while its water extracts has been shown to possess anti-metastatic activity by inhibiting the migration of colon carcinoma cells to lungs [58]. Two compounds isolated from *H. erinaceus* were demonstrated to possess anti-cancer activity in pre-clinical mouse models. The cyanthine diterpenoid Erinacine A has shown inhibition of DLD-1 human colorectal adenocarcinoma xenograft tumor growth in nude mice [59]. A protein isolated from *H. erinaceus* called HEP3 also showed ability to inhibit growth of colon cancer cells in mouse xenograft tumors [60]. Animal studies have also been conducted with *I. obliquus*, *Trametes versicolor* and *Phellinus linteus*. A proteoglycan isolated from *P. linteus* was able to potentiate immune system as well as inhibit cancer in HT-29-bearing BALB/c-nu/nu mice by inhibiting Reg IV/EGFR/Akt signaling pathway [61]. As for *I. obliquus*, its extracts were capable of inhibiting tumor growth in human melanoma B16-F10 cells- and sarcoma-180 cells-derived xenografts in mice [62,63]. Specific compounds isolated from *I. obliquus* have shown anti-cancer activity in vivo. Two unique lanostane-type, inonotodiol and inonotsuoxides, have demonstrated anti-carcinogenic effects on mouse skin and human leukemia-derived mouse xenograft tumors [64,65]. For *T. versicolor*, the β–glucan-based polysaccharopeptide fraction PSP exhibited growth-inhibitory effect on a number of cancer cells in mouse xenograft tumors [11]. In addition, a new glucan isolated from the water extract of *T. versicolor* was able to inhibit the xenograft sarcoma growth in mice [66].

#### 3.2.3. Anti-Proliferative/Anti-Cancer Activity in Humans

To date, there has been no clinical studies evaluating the anti-cancer potential of mushrooms native to North America. However, there are 600 published papers and reports of clinical therapeutic effects of medicinal mushrooms collected from different parts of the world. Here, we highlight some of the key clinical studies. Perhaps, the most clinically-relevant anti-cancer compounds from mushroom are PSP and polysaccharide-K (PSK). These immunotherapeutic anti-cancer compounds had been extensively studied clinically and have been in use in Japan since 1977 and in China since 1987 [11]. Another mushroom that has been studied extensively on humans is *G. lucidum*, commonly known as Reishi or Ling Zhi in Asia [42]. One such clinical study was conducted on Ganopoly, a polysaccharide isolated from *G. lucidum*. A randomized controlled double blinded clinical trial on 68 patients with advanced lung cancer was conducted to evaluate the efficacy and safety of Ganopoly. Patients were evaluated after 12 weeks of administration of Ganopoly. Ganopoly was found to enhance the host immune function by increasing the activity of macrophages, T lymphocytes and natural killer cells, suggesting its role as an adjunct [67]. Another study was conducted on 47 patients with advanced colorectal cancer in which 5.4 g/day doses of Ganopoly was administered to patients for 12 weeks. Ganopoly was found to stimulate the immune response in the cancer patients [68].

A phase I/II dose escalation trial has been carried out using polysaccharide extract from *G. frondosa*, also known as Maitake mushroom. The efficacy of the polysaccharide extract was evaluated at multiple doses ranging from 0.1–6 mg/kg two times daily for 3 weeks. Thirty postmenopausal breast cancer patients were enrolled and were sub-divided into 5 cohorts with 6 patients in each cohort. The polysaccharide extract stimulated the immune response at a dose of 6mg/kg [69]. A hospital-based case controlled study was conducted to assess the combined effect of edible mushrooms and green tea on breast cancer. The study showed a reduced risk of breast cancer in both pre- and postmenopausal breast cancer patients due to high intake of *A. bisporus*, *L. edodes* and green tea [70]. Another hospital-based case-controlled study showed a reduced risk of stomach cancer from higher intake of mushrooms including *Flammulina velutipes, Hypsizigus marmoreus, Corrinellus shiitake* and *Pholiota nameko* [71]. Lastly, a superfine dispersed lentinan (SDL) oral formulation was evaluated on advanced colorectal cancer. Lentinan is an immunomodulatory β-glucan isolated from *L. edodes.* Eighty patients with advanced colorectal cancer were administered with 15 mg dose per day of SDL for 12 weeks. The SDL formulation, found to be safe and efficacious, reduced the adverse effects of chemotherapy and was associated with increasing the quality of life of patients with advanced colorectal cancer [72].

### 3.3. Anti-Inflammatory Activity

#### 3.3.1. Anti-Inflammatory Activity In-Vitro

Mushrooms native to North America have also been explored for anti-inflammatory activity. Out of 29 species examined from north-central BC [24] and Haida Gwaii [23], 26 species (69%) had their anti-inflammatory activity reported for the first time. These species include *A. augusta, C. tomentosus, Clavulina cinerea, G. helvelloides, G. esculenta, Hydnellum repandum, Hygrophoropsis aurantiaca, Hypholoma fasciculare, Inocybe sp., L. conifericola, L. connate, Phellinus nigricans, P. atratus, P. ostreatus, R. cystidiophora, R. paludosa, T. abietinum, T. rutilans,* and *T. chioneus*. Extracts from *I. obliquus* collected in BC, like those found in other parts of the world, showed strong anti-inflammatory activity in vitro. Polymyxin B was used as positive control to assess anti-inflammatory activity [23,24]. In another study conducted on North American mushrooms, 95% ethanol extracts of *Elaphomyces granulatus, Rhizopogon nigrescens*, and *Scleroderma laeve* possessed strong anti-inflammatory activity whereas *E. muricatus, Hymenogaster subalpinus, Melanogaster tuberiformis, Rhizopogon couchii, R. subaustralis,* and *R. subgelatinosus* were also active against inflammation. COX-2 activity was assessed to determine the anti-inflammatory potential and NS-0398 was used as a positive control [25]. A limited number of anti-inflammatory compounds have been isolated from mushrooms native to North America. A 5 kDa polysaccharide isolated from the NaOH extract of North American mushroom *E. tinctorium* was able to induce anti-inflammatory response in Raw264.7 macrophage cells [18]. A polysaccharide called CDP from *Gymnopus dryophilus* [31], and CDP-like polysaccharides from *L. edodes* and *Marasmius oreades* [32], can inhibit nitric oxide production in Raw264.7 macrophage cells. Elsewhere, the ethanolic extract as well as two small molecules, syringaldehyde and syringic acid, isolated from *E. granulatus* inhibited COX-2 enzyme in Raw264.7 cells [29]. The extract caused 68% inhibition of COX-2 at 50 μg/mL, whereas syringaldehyde and syringic acid were effective with IC50 of 3.5 μg/mL and 0.4 μg/mL respectively. The COX-2 inhibition of small molecules was compared with a positive control, NS-398, a known COX-2 inhibitor with IC50 of 0.2 μg/mL (0.64 μM) [29].

#### 3.3.2. Anti-Inflammatory Activity in Animal Models

Currently, there are very limited studies examining the anti-inflammatory potential of North American mushrooms using animal models. In one study, the polysaccharide AIPetinc isolated from *E. tinctorium* showed anti-inflammatory activity on histamine-induced inflammatory mouse microcirculation model (*n* = 10 mice) [18]. An intra-vital mouse model was used to determine acetylcholine-induced vasodilation, which is one of the events in inflammation. AIPetinc was able to induce nitric oxide production, reversed the response to histamine and restored the vascular integrity, thereby resulting in anti-inflammatory response [18]. To determine the significance of the anti-inflammatory activity of AIPetinc, it should be further evaluated in other animal models. In another study, the methanol extracts of *I. obliquus* was found to attenuate histamine-induced inflammation conducted vasodilation in arterioles in the gluteus muscle of mice (*n* = 41) [34]. Anti-inflammatory compounds ergosterol, ergosterol peroxide and trametenolic acid had been isolated from the sclerotia of *I. obliquus* available from Haerbin, China [73]. It is unknown whether these compounds are present in the wild *I. obliquus* from B.C. and were responsible for attenuating histamine-induced inflammation in the gluteus muscle mouse model [34].

#### 3.3.3. Anti-Inflammatory Activity in Clinical Studies

Given that there had been only two animal studies as described above, it is not surprising that there are currently no clinical studies examining anti-inflammatory potential of mushrooms North America. However, we wish to draw readers’attention to another review article that has summarized clinical studies alongside in-vitro and animal studies on investigating anti-inflammatory properties of mushrooms [74].

### 3.4. Immuno-Stimulatory Activity

#### 3.4.1. *Immuno-Stimulatory* Activity In-Vitro

Mushroom species are known to have immunotherapeutic properties and over 270 species had been recognized for such medicinal potential [75]. Mushrooms are natural immune modulators that can enhance the host immune system by activating dendritic cells, T cells, NK cells, macrophages, and cytokines. Mushroom native to North America have also been explored for immuno-stimulatory activity. Out of 29 species of mushrooms examined from north-central BC [24] and Haida Gwaii, BC [23], 20 species exhibited immuno-stimulatory activity. Fifteen out of the 20 species (75%) had their immuno-stimulatory activity reported for the first time [23,24]. These species include *A. augusta, C. tomentosus, C. cinerea, F. fomentarius, G. helvelloides, G. esculenta, Hericium corralloides, H. repandum, Hydnellum* sp.*, H. aurantica, H. fasciculare, Inocybe* sp*., L. conifericola, L. connata, P. nigricans, P. atratus,* and *Piptoporus betulinus*.

To date, most of the immuno-stimulatory compounds from mushrooms are polysaccharides or polysaccharide-protein complexes [76,77]. Due to problems such as: (i) high variability of polysaccharides that is further exacerbated during extraction and purification, (ii) impurities and contaminants, and (iii) difficulty in defining the structure and chemical fingerprint to understand structure-function relationships [78], there has been little interest amongst scientists in North America to study and isolate immuno-stimulatory polysaccharides from mushrooms native to North America.

#### 3.4.2. *Immuno-Stimulatory* Activity in Animal Models

There is currently no animal studies examining the immuno-stimulatory activity of mushrooms native to North America. However, there were many animal studies evaluating the immuno-stimulatory effects of mushrooms collected elsewhere [11]. For instance, there were animal studies examining immunomodulatory activity of *G. lucidum* and its related species, *G. tsugae* and *G. applanatum* [79]. In another study, Agarikon 1 and Agarikon Plus which contains medicinal mushroom mixtures, were found to activate and maintain the immune system equilibrium in an advanced colorectal mouse model [80]. Elsewhere, the hot water, methanol and polysaccharide extracts of *Lentinula edodes* induced immune response against eimeriasis in chicken [81].

#### 3.4.3. *Immuno-Stimulatory* Activity in Clinical Studies

As with animal studies, there is currently no clinical studies evaluating the immuno-stimulatory effect of North American mushrooms. There are also limited clinical studies evaluating immuno-potentiating effects of mushrooms collected from elsewhere. Two of the most notable medicinal mushrooms. *T. versicolor* and *Grifola frondosa* (Maitake mushroom), had been clinically investigated for such purposes. As described in 3.2.3, both PSP and PSK are immuno-potentiating anticancer polysaccharides isolated from *T. versicolor* that had undergone many clinical studies and this has been critically reviewed recently [11]. For *G. frondosa*, its polysaccharide extract was administered to 34 breast cancer patients on a phase I/II clinical trial. Cytokine analysis in patients’ peripheral blood showed signs of both immuno-stimulatory and –inhibitory effects [82]. There was no dose-limiting toxicity observed in patients, suggesting that the extract is safe. The authors concluded that the *G. frondosa* polysaccharide should be classified as immuno-modulatory rather than immuno-stimulatory.

### 3.5. Anti-Oxidant Activity

#### 3.5.1. Anti-Oxidant Activity In-Vitro

There has been relatively less study on anti-oxidant activity of mushrooms native to North America. One study reported ethanolic extracts from 11 species of mushrooms collected from Idaho, Oregon, and New Jersey, to have moderate to weak anti-oxidant activity. In the study, 70–95% ethanolic extracts of various mushrooms were assessed and Vitamin C was used as a positive control [25]. *Scleroderma laeve* showed strong anti-oxidant activity with an IC50 of less than 20 μg/mL whereas *Rhizopogon pedicellus*, *R. couchii, Leucogaster rubescens,* and *Geopora clausa* had moderate anti-oxidant effect with IC50 of 20–50 μg/mL. *Elaphomyces granulatus, E. muricatus, Gautieria monticola, Melanogaster tuberiformis, Rhizopogon nigrescens,* and *R. subaustralis* had weak anti-oxidant activity with IC_50_ values of more than 50 μg/mL [25].

The ethanolic extract as well as syringic acid isolated from the fruiting bodies of *Elaphomyces granulatus* showed potent antioxidant effect on myelomonocytic HL-60 cells with an IC50 of 41 μg/mL for the extract and 0.7 μg/mL for syringic acid. Vitamin C was used as a positive control with IC50 of 0.5 μg/mL (2.84 μM) to compare the anti-oxidant activity of syringic acid and ethanol extracts [29]. This study shows the promising effects of syringic acid as a potent anti-oxidant and can be considered for drug development. Two edible mushrooms, *P. ostreatus* from USA and *P. levis* from Mexico, also showed antioxidant activity. The effective concentration (EC-50) was also determined via DPPH free radical scavenging assay, ABTS scavenging assay, and β-carotene linoleic acid assays where BHA, BHT, α-tocopherol, and ascorbic acid were used as standards [39]. The anti-oxidant activity was also determined by oxygen radical absorbance capacity assay and the anti-oxidant effect was compared with a known anti-oxidant, Trolox, a vitamin E analog [39].

#### 3.5.2. Anti-Oxidant Activity in Animal Models

As with the immuno-stimulatory activity, there is currently no study on the evaluation of North American mushrooms for anti-oxidant activity in animal models. However, there were animal studies investigating the anti-oxidant effect of medicinal mushrooms collected elsewhere. For instance, since oxidative stress plays important role in the etiology of diabetes, the impact of mushrooms on antioxidant enzymes involved in streptozotocin-induced diabetes were investigated in mouse models [83,84]. The effect of administration of G. lucidum and Agaricus brasiliensis were studied in rat leukocytes. Streptozotocin was administered intraperitoneally at 50 mg/kg body weight to induce diabetes whereas mushrooms were given orally at a dose of 1 g/kg every day for 2 weeks. Mushroom preparations were found to increase the activity of anti-oxidant enzymes [83]. Another study also determined the anti-oxidant enzymes protection by mycelia of the two mushrooms on rat erythrocytes after induction of diabetes by streptozotocin [84]. In another separate study, anti-oxidant effect and regulation of lipid metabolism by *F. velutipes* extracts was studied. Forty eight hamsters were divided into eight groups, six hamsters/group, where one group was given normal diet while others were given high fat diet. 1–3% of mushroom preparation was added to their diet for 8 weeks, which resulted in significant effect on lipid metabolism of hamsters with high fat diet [85].

### 3.6. Anti-Fungal Activity

Many mushrooms are known to possess antifungal activity [86]. However, there has been only one study on anti-fungal activity of mushroom native to North America. A study shows that a chlorinated orcinol derivative, 2-chloro-1,3-dimethoxy-5-methyl benzene isolated from mycelial cultures of *Hericium* sp. collected in Minnesota (USA), showed inhibitory effect on *Candida albicans* and *Candida neoformans*, suggesting its role as an antifungal agent [17]. *Hericium sp*. is an edible mushroom containing multiple small molecules, making it an attractive option for further screening.

### 3.7. Other Bioactivities

In addition to the bioactivities described above, mushrooms from North America have also been investigated for antiparasitic, antimalarial and antituberculosis activities. A study conducted in Mexico showed antiparasitic effects of hydroalcoholic extracts from *Pleurotus djamor* against *Haemonchus contortus* eggs [38], suggesting its metabolites can act as anthelmintics. In the same study, few small molecules with antiparasitic activity were also isolated [38]. Stanikunaite and co-workers assessed 22 species of mushrooms native to North America and found *Rhizopogon subareolatus* to have antimalarial activity [25]. They also found the following species to have antituberculosis activity: *Astraeus pteridis, Barssia oregonensis, Elaphomyces granulatus, Elaphomyces muricatus, Hymenogaster subalpinus, Melanogaster tuberiformis, Rhizopogon couchii, Rhizopogon pedicellus, Rhizopogon subareolatus,* and *Scleroderma leave* [25].

## 4. Bioactive Compounds from Mushrooms Native to North America

**Figure 1:**
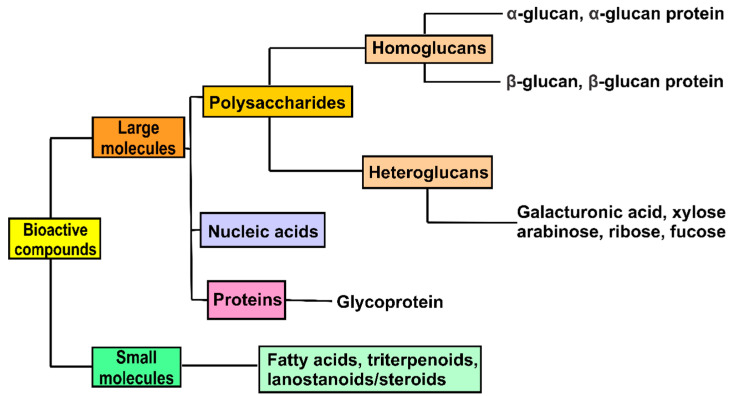
Classification of bioactive compounds isolated from mushrooms.

Since there is relatively limited number of studies on mushrooms native to North America, it is not surprising to find relatively limited number of bioactive compounds isolated from the mushrooms. Here, we summarize all the bioactive compounds that have been isolated from mushrooms native to North America into two general groups, large molecules and small molecules (Table 2) (Figure 1).

### 4.1. Large Molecular Weight Compounds

Large molecular weight compounds isolated from mushrooms are typically homo- and heteroglycans, proteins, polysaccharide-protein complexes and nucleic acids-protein complexes (Figure 1) [87]. Table 2 summarizes the bioactive large molecules that have been isolated from North American wild mushrooms. A 229 kDa growth-inhibitory heteroglycan GIPinv was isolated from the 5% NaOH extract of *P. involutus* [87]. GIPinv is made up predominantly of glucose (65.9%), galactose (20.8%) and mannose (7.8%) with traces of fucose (3.2%) and xylose (2.3%) [31]. It has mixed linkages in the backbone containing (1→6)-Gal, (1→4)-Glc, (1→6)-Glc, (1→3)-Glc, and (1→2)-Xyl, with branching points at (1→2,6)-Man and (1→3,6)-Man. Another growth-inhibitory polysaccharide, an exopolysaccharide called EPS, was isolated from *P. tuber-regium* [51]. EPS is 3,180 kDa and consisted mainly of mannose (57.5%) and glucose (42.5%). Another study showed anti-proliferative effect of a 12 kDa 130-amino acid containing glycan binding protein (Y3). Y3 is a tertiary protein isolated from *Coprinus comatus* and exhibited selective anti-proliferative effects in human T cell leukemia Jurkat cells [28].

A 1234 kDa anti-inflammatory β-glucan polysaccharide CDP consisting of (1→3) and (1→4) glucosidic linkages was isolated from aqueous extract of the fruiting bodies of *G. dryophilus* [31,32]. The authors treated mouse macrophage Raw 264.7 cells with 50 to 1000 μg/mL of CDP and showed that it had no toxicity effects [32]. Some other CDP-like polysaccharides 610 kDa and 1316 kDa in size with anti-inflammatory potential were isolated from the aqueous extracts of *L. edodes* and *Marasmius oreades* respectively [32]. In the same study, water-soluble CDP-like polysaccharides were also isolated from multiple mushrooms obtained from Quebec (Canada). These include *Agaricus arvensis*, *Tricholoma flavovirens*, *Suillus americanus*, *Amanita muscaria*, *Amanita rubescens*, *Lycoperdon pyriforme*, *Coprinus atramentarius*, *C. comatus*, *Hydnum imbricatum*, *Lactarius deliciosus*, *Leccinum aurantiacum*, *Leccinum subglabripes*, *Lepiota americana*, *Panellus serotinus*, *P. betulinus*, *Polyporus squamosus*, *Russula variata*, and *T. vaccinum* [32]. Another relatively small 5 kDa β-glucan called AlPetinc with anti-inflammatory activity was isolated from the 5% NaOH extract of *E. tinctorium* [18]. AlPetinc is a heteroglucan composed mainly of glucose (88.6%) with a small amount of mannose (4.4%), galactose (4.0%), xylose (2.3%), and fucose (0.7%).

As mentioned briefly in Section 3.4, there has been a lack of interest amongst scientists in the West, especially in North America, in studying bioactive polysaccharides for use as medicinal compounds [78]. However, with recent advances in identifying biosynthetic gene clusters and transcriptomic studies including those in fungi, it is now possible to produce compounds including large polysaccharides using heterologous expression systems by genetic engineering [88,89,90,91]. Such efforts are expected to enable large scale production of pure bioactive polysaccharides, thereby overcoming some if not all of the problems previously encountered [78].

### 4.2. Small Molecules

**Figure 2:**
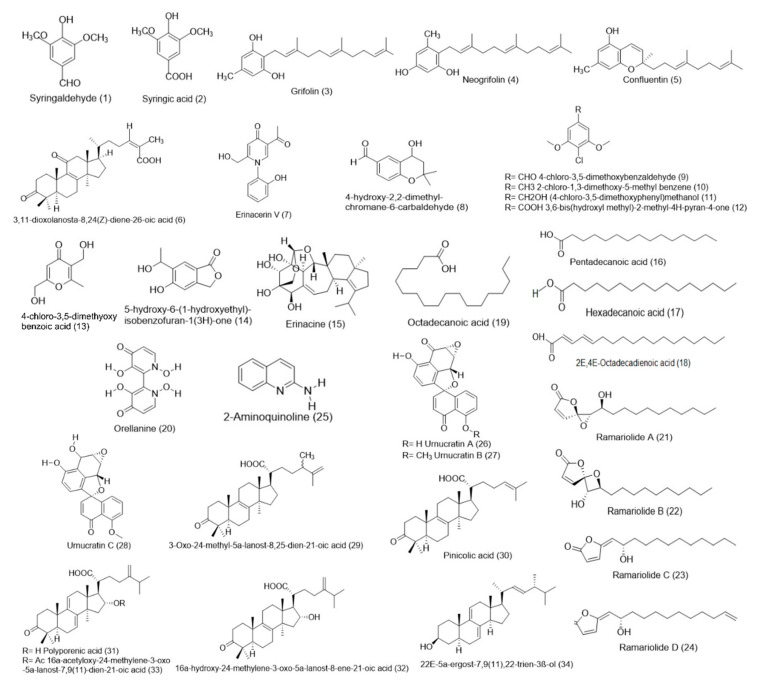
Structures of small molecules isolated from mushrooms native to North America.

Small molecules that had been isolated from mushrooms include quinones, cerebrosides, amines, catechols, sesquiterpenes, triacylglycerols, steroids, organic germanium, and selenium (Figure 1) [87]. Amongst the handful of small molecules isolated from North American mushrooms, few are terpene derivatives as shown in Table 2 and Figure 2.

Two small molecules, syringaldehyde and syringic acid with molar mass of 182 g/mol and 198 g/mol respectively, were isolated from ethanol extract of the fruiting bodies of *E. granulatus* [29]. The possible toxicity of both compounds was evaluated on macrophage Raw 264.7 cells and HL-60 cells. The authors found that both syringaldehyde and syringic acid had no effect on Raw 264.7 and HL-60 cells viability of up to 25 μg/mL and 31.25 μg/mL respectively [29]. Grifolin (328 g/mol), neogrifolin (328 g/mol) and confluentin (326 g/mol) with growth-inhibitory and antibacterial activities were isolated from the ethanol extract of the fruiting bodies of *A. flettii* [21,22]. A lanostane-type triterpene named 3, 11-dioxdanosta-8,24(Z)-diene-26-oic acid with molar mass of 468 g/mol was isolated from *J. hirtus*. This triterpene effectively inhibited the growth of two Gram positive bacteria; *Bacillus cereus* and *Enterococcus faecalis* [21]. Three new bisnaphthalene subclass compounds called urnucratins A–C were isolated from the North American cup fungus *U. craterium* [41]. Urnucratin A (348 g/mol) was found to be active against methicillin-resistant *Staphylococcus aureus*, vanocymin-resistant *Enterococcus faecium* and *Streptococcus pyogenes* [41]. A new lanostane triterpenoid named 3-oxo-24-methyl-5α-lanost-8,25-dien-21-oic acid (468 g/mol) with activity against *B. cereus* was isolated from *F. pinicola* native to North America [30]. The same study also reported isolation of four known lanostane triterpenoids and one known ergostane steroid with anti-bacterial activity [30].

Many small molecules were isolated from *Hericium* sp. which included two new compounds, an erinacerin V alkaloid with molar mass of 257 g/mol and an aldehyde derivative of 4-hydroxychroman, 4-chloro-3,5-dimethoxybenzaldehyde with molar mass of 206 g/mol [17]. Seven known compounds were also isolated from *Hericium* sp. which included 2-chloro-1,3-dimethoxy-5-methylbenzene, (4-chloro-3,5-dimethoxyphenyl) methanol, 3,6-bis(hydroxymethyl)-2-methyl-4*H*-pyran-4-one, 4-chloro-3,5-dimethoxybenzoic acid, 5-hydroxy-6-(1-hydroxyethyl)isobenzofuran-1(3*H*)-one, and erinacine [17]. Some other small molecules that had been isolated from North American mushrooms include pentadecanoic acid, hexadecanoic acid, 2*E*,4*E*-octadecadienoic acid, and octadecanoic acid from *Pleurotus djamor* [38], and orenalline from *Cortinarius armillatus* [19]. Most of these compounds have not been assessed for bioactivity and toxicity except orenalline. There were many studies on evaluating the toxicity of orenalline which is considered a nephrotoxin [20]. Four new compounds belonging to the butenolide groups called ramariolides A–D were isolated from the coral mushroom *Ramaria cystidiophora* collected in southwestern British Columbia (Canada) [50]. Ramariolides A was found to have antimicrobial activity against *Mycobacterium smegmatis* and *Mycobacterium tuberculosis* [50].

## 5. Mushrooms from North America as a Source for Drug Discovery

As shown in Table 1, to date 79 species of mushrooms native to North America had been investigated and exhibited medicinal properties. Out of these, 48 species (60%) have bioactivities that have not been previously reported (Table 1). These include *A. augusta* with strong anti-proliferative activity, *G. dryophilus* with anti-inflammatory activity, *G. esculenta* with strong immunostimulatory activity, and *H. coralloides* with strong immuno-stimulatory activity [23,24]. This also includes *C. tomentosus, G. helvelloides, L. conifericola, L. connata, T. abietinum* with strong activity in antiproliferation, immunostimulation and anti-inflammation [23,24]. There were specimens that could only be identified to the genus level, *Inocybe* sp. and *Hydnellum* sp., suggesting that they are new species. *Inocybe* sp. showed strong anti-proliferative, immuno-stimulatory and anti-inflammatory activities, while *Hydnellum* sp. exhibited strong anti-proliferative activity [24]. There were 13 species that had been studied elsewhere, but their new bioactivities were discovered in the species collected in North America (Table 1). This includes *Haploporus odorus* with antimicrobial activity; *C. cinerea, H. repandum, H. aurantiaca, H. fasciculare, P. nigricans,* and *P. ostreatus* with anti-inflammatory activity; *R. cystidiophora, R. paludosa, T. rutilans,* and *T. chioneus* with anti-proliferative and anti-inflammatory activities.

As shown in Table 2, there has been very limited number of studies exploring bioactive compounds from North American mushrooms. Despite the limited number of studies, 10 new small molecules, six new polysaccharides and a new protein with bioactivity have been discovered (Table 2).

Such observations and the fact that a large number of mushrooms native to North America having bioactivities that have never been previously described, strongly suggests that North American mushrooms are indeed a good source for drug discovery.

## 6. Edibility of Mushrooms Native to North America

North American mushrooms offer a wide variety of medicinal benefits and some have been appreciated for their exquisite flavors. While some are unique and edible, others are toxic for human consumption. Edible mushrooms are valuable as functional foods. Nonetheless, both edible and non-edible mushrooms offer great benefits against diseases. Edibility of mushrooms is an important attribute that allows them to be used as functional foods and potential phytomedicines. Like elsewhere, many edible mushrooms are found in North America. Here, we only highlight some of the popular ones. Some of the North American edible mushrooms [92,93] with medicinal properties are *A flettii, I obliquus (Chaga), G. dryophilus, C. comatus, L. edodes* (Shiitake)*, Marasmius oreades* (fairy ring mushroom)*, Boletus curtisii,* and *Amanita augusta*. Most mushrooms that belong to the *Pleurotus* genus are also edible and they include *P. tuberregium*, *P. djamor, P. ostreatus*, and *P. levis*. *G. helvelloides* is edible but it is rather bland and is therefore not much preferred in culinary. One of the most popular wild edible mushrooms in North America is chanterelle [85]. There are over 70 species of chanterelles (*Cantharellus* spp.) described worldwide and the most sought-after in North America is the Pacific golden chanterelle (*Cantharellus formosa*) found from northern California to northern BC [94]. Another popular culinary mushroom in North America as well as in Europe is *Morchella*, the true morels. At least 19 species of *Morchella* had been identified in North America [95] with black morels (*Morchella elata* and related species) found in Washington State and BC being one of the most highly demand culinary mushrooms. Lastly, the wild edible lobster mushroom (*Hypomyces lactifluorum/Russula brevipes*), uniquely confined to North America, is not a species but the result from infection of *Russula* (mostly *Russula brevipes*) or *Lactarius* genera by *Hypomyces lactifluorum* parasite [96]. The lobster mushroom, widely distributed in northern parts of United States and in Canada, has a seafood-like flavor and is enjoyed by many locals.

North American medicinal mushrooms that are considered toxic or non-edible [92,93] for general human consumption can be tailored as drugs after sufficient toxicity testing and targeted dosage form development. Some of the features that make mushrooms inedible are the presence of toxic metabolites in the whole mushroom, its taste and toughness. *E. tinctorium* is a hard, inedible conk. *P. atratus* and *J. hirtus* are also tough mushrooms and apparently *J. hirtus* has bitter taste. *P. involutus* is considered poisonous for human use. *E. granulatus* is also non-edible. At high doses, *C. armillatus* is considered toxic due to the presence of orenalline [19], a potent nephrotoxin originally isolated from *Cortinarius orellanus* [97]. Therefore, it is possible that orenalline is present at low amount in *C. armillatus.*

## 7. Conclusions

Having extensively reviewed the literature on the bioactivities and compounds isolated from mushrooms native to North America, we can now attempt to answer the questions posed at the beginning of this review. (i) Do similar species found in North America and elsewhere exhibit similar bioactivities and produce similar group of bioactive compounds? Based on subjective approach of bioactivity-guided investigations on selective species, similar species found in North America and elsewhere indeed produce similar bioactivities. For example, this is true for the commonly *studied I. obliquus and P. ostreatus*. Furthermore, compounds such as grifolin, neogrifolin and confluentin produced in North American *A. flettii*, are also made by other *Albatrellus* genus found elsewhere [21,22]. However, a better answer can only be obtained by using more advanced and objective method such as Quadruple Time-of-Flight Mass Spectrometry to globally examine the metabolites produced and using multiple biological screening assays to simultaneously monitor different bioactivities. (ii) Do similar species found in North America and elsewhere exhibit distinct bioactivity and produce distinct compounds? Again, the answer to this question would come from more advanced approaches described above. (iii) Do new species found in North America produce new compound(s)? Due to limited number of studies on mushrooms native to North America, it is currently unknown whether new species found in North America produce new compound(s). However, based on very limited study (Table 2), it is likely that new medicinal compounds are produced by new species found in North America. (iv) Based on the answers to (i) to (iii) above, is it worth exploring mushrooms native to North America for medicinal properties and for new compounds? We believe that North American mushrooms are worth further exploration. This is based on the fact that there are many species, including new species, in North America whose bioactivities have never been previously reported (Table 1). Furthermore, novel compounds have been isolated from North American mushrooms despite the very limited number of studies (Table 2).

In summary, to date only 79 mushroom species in North America that have been studied and reported to possess medicinal properties. Of these, 48 species (60%) exhibited bioactivities that have never been previously reported (Table 1). To date, only 16 mushroom species in North America that had been subjected to bioactivity-guided compound isolation studies. Out of this limited number of studies, already 10 new small molecules, 6 new polysaccharides and a new protein with medicinal properties have been discovered (Table 2). For the majority of these compounds, studies are still at an early stage using cell lines, and their significance needs to be demonstrated in animal models and beyond.

## Figures and Tables

**Table 1:** Mushrooms Native to North America Studied for Bioactivities.

Mushroom Species	Origin	Bioactivity	Bioactive Component ^1^ or Extraction Solvent	Active Dose ^2^
*Albatrellus flettii*	La Crosse, Wisconsin	Antimicrobial [21]	Grifolin (**3**) Neogrifolin (**4**) Confluentin (**5**)	MIC = 10 μg/mL (**3**), 20 μg/mL (**4**,**5**) on B. cereus. MIC = 0.5 μg/mL (**3**,**4**), 1 μg/mL (**5**) on E. faecalis
*Albatrellus flettii*	Smithers, BC	Anti-proliferative [22]	Grifolin (**3**) Neogrifolin (**4**) Confluentin (**5**)	IC_50_ = 27.4 μM (3), 24.3 μM (4), 25.9 μM (5) on HeLa. IC_50_ = 35.4 μM (**3**), 34.6 μM (4), 33.5 μM (**5**) on SW480. IC_50_ = 30.7 μM (**3**), 30.1 μM (**4**), 25.8 μM (**5**) on HT29.
*Amanita augusta*	Haida Gwaii, BC	Anti-proliferative [23]	50% methanol (a) and 5% NaOH (b)	IC_50_ = 0.7 mg/mL (a), 0.55 mg/mL (b)
*Amanita augusta*	Haida Gwaii, BC	Immuno-stimulatory [23]	Water	1 mg/mL
*Amanita augusta*	Haida Gwaii, BC	Anti-inflammatory [23]	5% NaOH	1 mg/mL
Amanita muscaria	Prince George, BC	Anti-proliferative [24]	80% ethanol (a) and 50% methanol (b)	IC_50_ = 0.2 mg/mL (a) (+++) IC_50_ = 0.6 mg/mL (b) (++)
Amanita muscaria	Prince George, BC	Immuno-stimulatory [24]	2% ammonium oxalate	1 mg/mL (+)
Astraeus pteridis	Linn County, Oregon	Antituberculosis [25]	95% ethanol	IC_50_ = < 20 μg/mL (+++)
Auricularia fuscosuccinea	Oxford, Ohio	Antimicrobial [26]	Water	Against B. subtilis (+++), P. aeruginosa, S. epidermidis (MIC = 1 mg/mL), P. fluorescens, M. luteus (++)
Barssia oregonensis	Clackamas, Oregon	Antituberculosis [25]	95% ethanol	IC_50_ = 20–50 μg/mL (++)
Cantharellus cibarius	Haida Gwaii, BC	Anti-proliferative [23]	80% ethanol	0.1 mg/mL
Cantharellus cibarius	Haida Gwaii, BC	Weak immuno-stimulatory [23]	Water	1 mg/mL
Cantharellus cibarius	Haida Gwaii, BC	Anti-inflammatory [23]	80% ethanol, 50% methanol, water and 5% NaOH	1 mg/mL
Chroogomphus tomentosus	Haida Gwaii, BC	Anti-proliferative (a), immuno-stimulatory (b) [23]	80% ethanol (a) and water (b)	0.2 mg/mL (a) (++), 1 mg/mL (b) (++)
Chroogomphus tomentosus	Haida Gwaii, BC	Anti-inflammatory [23]	80% ethanol (a) and 50% methanol (b)	1 mg/mL (a,b) (+++)
Clavulina cinerea	Haida Gwaii, BC	Anti-proliferative [23]	80% ethanol	(++)
Coprinellus sp.	Seattle, WA, USA	Anti-proliferative [27]	Water	IC_50_ = 40 μg/mL on MDA-MB-231 cells (+++), 120 μg/mL on MCF-7 cells (+), 150 μg/mL on BT-20 cells (+)
Coprinus comatus	Seattle, WA, USA	Anti-proliferative	Water (a) [27], Protein (b) [28]	IC_50_ = 400 μg/mL on MDA-MB-231 cells (a), 450 μg/mL on MCF-7 cells (a), 10 μM (b)
*Cortinarius armillatus*	Massachusetts	Anti-proliferative [19]	Orellanine	20 μg/mL clear cell RCC (a), 40 μM (10 mg/L) intraperitoneal injection via PD solution (b)
*Echinodontium tinctorium*	Smithers and Terrace, BC	Anti-inflammatory [18]	Polysaccharide AIPetinc	1 mg/mL
Elaphomyces granulatus	Oregon and Bonner County, Idaho	Anti-inflammatory [25,29]	Syringaldehyde (**1**), Syringic acid (**2**), and 95% ethanol (a)	IC_50_ = 3.5 μg/mL (**1**) (19.23 μM) (+) IC_50_ = 0.4 μg/mL (**2**) (2.02 μM) (+++) 50 μg/mL (a) (+++)
Elaphomyces granulatus	Oregon and Bonner County, Idaho	Antioxidant [29]	Syringic acid (**2**) and 95% ethanol	IC_50_ = 0.7 μg/mL (2) (+++) Extract IC_50_ = 41 μg/mL
Elaphomyces muricatus	Benton County, Oregon	Anti-inflammatory (a), antioxidant (b), antituberculosis (c) [25]	95% ethanol (a,c), 70% ethanol (b)	50 μg/mL (a) (++), IC_50_ ≥ 50 μg/mL (a) (+), IC_50_ ≥ 50 μg/mL (a) (+)
Flammulina velutipes	Seattle, WA, USA	Anti-proliferative [27]	Water	IC_50_ = 30 μg/mL on BT-20 cells (+++), 75 μg/mL on MDA-MB-231 cells (++), 150 μg/mL on MCF-7 cells (+)
*Fomes fomentarius*	Prince George, BC	Anti-proliferative [24]	80% ethanol (a) and 50% methanol (b)	0.5 mg/mL (a) (+++), 0.5 mg/mL (b) (+++)
*Fomes fomentarius*	Prince George, BC	Immuno-stimulatory [24]	Water (a) and 2% ammonium oxalate (b)	1 mg/mL (a) (+), 1 mg/mL (b) (++)
*Fomitopsis pinicola*	Oregon, USA	Antimicrobial [30]	3-Oxo-24-methyl-5α-lanost-8,25-dien-21-oic acid (**30**), pinicolic acid (**31**), polyporenic acid (**32**), 16α-hydroxy-24-methylene-3-oxo-5α-lanost-8-ene-21-oic acid (**33**), 16α-acetyloxy-24-methylene-3-oxo-5α-lanost-7,9(11)-dien-21-oic acid (**34**), 22E-5α-ergost-7,9(11),22-trien-3β-ol (**35**)	MIC = 32 μg/mL (**30**) (++), 16 μg/mL (**31**) (++), 32 μg/mL (**32**) (++), 32 μg/mL (**33**) (++), 128 μg/mL (**34**) (+), 64 μg/mL (**35**) (++) against B. cereus
Ganoderma applanatum	Terrace, BC and Oxford, Ohio	Anti-proliferative [24]	80% ethanol (a) and water (b)	0.5 mg/mL (a) (++), 1 mg/mL (b) (+)
Ganoderma applanatum	Terrace, BC and Oxford, Ohio	Anti-inflammatory [24]	80% ethanol (a) and 50% methanol (b)	1 mg/mL (a) (+++), 1 mg/mL (b) (+++)
Ganoderma applanatum	Terrace, BC and Oxford, Ohio	Immuno-stimulatory [24]	Water(a), 5% NaOH (b), and 2% ammonium oxalate (c)	1 mg/mL (a) (+++), 1 mg/mL (b) (+), 1 mg/mL (c) (+)
Ganoderma applanatum	Terrace, BC and Oxford, Ohio	Antimicrobial [26]	Water	Against P. aeruginosa, P. fluorescens, B. subtilis, S. epidermidis, (MIC = 100 mg/mL (isolate 1), 10 mg/mL (isolate 2), and M. luteus (+++)
Ganoderma lucidum	Oxford, Ohio	Antimicrobial [26]	Water	MIC = 0.1 mg/mL (+++) against S. epidermidis
Ganoderma tsugae	Haida Gwaii, BC	Anti-proliferative (a), immuno-stimulatory (b) [23]	80% ethanol (a) and water (b)	(a) (++),1 mg/mL (b) (+++)
Ganoderma tsugae	Haida Gwaii, BC	Anti-inflammatory [23]	80% ethanol (a) and 5% NaOH (b)	1 mg/mL (a) (+++), 1 mg/mL (b) (++)
Gautieria monticola	Benton County, Oregon	Antioxidant [25]	70% ethanol	IC_50_ = > 50 μg/mL (+)
Geopora clausa	Inyo Country, California	Antioxidant (a), anti-proliferative [25]	70% ethanol	IC_50_ = 20–50 μg/mL (a) (++)
Guepina helvelloides	Haida Gwaii, BC	Anti-proliferative (a), immuno-stimulatory (b) [23]	80% ethanol (a) and water (b)	(a) (++),1 mg/mL (b) (+++)
Guepina helvelloides	Haida Gwaii, BC	Anti-inflammatory [23]	80% ethanol (a), 50% methanol (b), and 5% NaOH (c)	
Gymnopus dryophilus	Quebec	Anti-inflammatory [31,32]	CDP polysaccharide	400 and 800 μg/mL
Gyromitra esculenta	Prince George, BC	Anti-proliferative [24]	80% ethanol, 50% methanol	1 mg/mL (+)
Gyromitra esculenta	Prince George, BC	Immuno-stimulatory [24]	80% ethanol, 50% methanol, water	1 mg/mL (++)
Haploporus odorus	Study conducted in Calgary, Canada	Antimicrobial [33]	Extracts	-
Hericium corralloides	Prince George, BC	Immuno-stimulatory [24]	50% methanol (a), water (b), 5% NaOH (c)	1 mg/mL (a) (++), 1 mg/mL (b) (++), 1 mg/mL (c) (+)
Hericium corralloides	Prince George, BC	Anti-inflammatory [24]	80% ethanol	1 mg/mL (+)
*Hericium* sp.	Minnesota	Antifungal [17]	2-chloro-1,3-dimethoxy-5-methyl benzene (10)	MIC = 31.3–62.5 μg/mL against *C. albicans* and *C. neoformans*
*Hericium* sp.	Minnesota	Antifungal (a), antibacterial (b) [17]	Ethyl acetate, acetone, methanol	MIC = 250 μg/mL against *C. albicans* and *C. neoformans* (a), MIC > 500 μg/mL) against *S. aureus* (b)
Hydnellum sp.	Prince George, BC	Anti-proliferative (a), immuno-stimulatory (b) [24]	80% ethanol, 50% methanol, water	1 mg/mL (a) (++), 1 mg/mL (b) (+)
*Hydnum repandum*	Haida Gwaii, BC	Anti-proliferative (a), anti-inflammatory (b & c) [23]	80% ethanol (a) (b) and 50% methanol (c)	0.6 mg/mL (a) (++), 1 mg/mL (b) (+++), 1 mg/mL (c) (++)
*Hygrophoropsis aurantiaca*	Haida Gwaii, BC	Anti-proliferative, [23]	50% methanol, water and 5% NaOH	(+)
*Hygrophoropsis aurantiaca*	Haida Gwaii, BC	Anti-inflammatory [23]	80% ethanol, 50% methanol, and water	1 mg/mL (+++)
*Hymenogaster subalpinus*	Benton County, Oregon	Anti-inflammatory (a), antituberculosis (b) [25]	95% ethanol	50 μg/mL (a) (++), IC50 = < 20 μg/mL (b) (+++)
*Hymenopellis furfuracea*	Oxford, Ohio	Antimicrobial [26]	Water	Against P. fluorescens, M. luteus (++), B. subtilis, S. episdermidis (+)
*Hypholoma fasciculare*	Haida Gwaii, BC	Anti-proliferative [23]	80% ethanol (a) and 50% methanol (b)	0.2 mg/mL (a) (++), 0.1 mg/mL (b) (++)
*Hypholoma fasciculare*	Haida Gwaii, BC	Anti-inflammatory [23]	80% ethanol, 50% methanol, and water	1 mg/mL (+++)
*Inocybe sp.*	Haida Gwaii, BC	Anti-proliferative, [23]	80% ethanol	(++)
*Inocybe sp.*	Haida Gwaii, BC	Immuno-stimulatory [23]	50% methanol and water	1 mg/mL (++)
*Inocybe sp.*	Haida Gwaii, BC	Anti-inflammatory [23]	80% ethanol (a) and 5% NaOH (b)	1 mg/mL (a) (+++), 1 mg/mL (b) (+)
*Inonotus obliquus*	Manitoba & Prince George, BC	Anti-inflammatory [34,35]	50% methanol	0.25 μg/μL (a), 1 μg/μL in vivo (b)
*Jahnoporus hirtus*	USA	Antimicrobial [21]	3,11-Dioxolanosta-8,24(*Z*)-diene-26-oic acid (6)	40 μg/mL (B. cereus), 32 μg/mL (E. faecalis)
*Laetiporus conifericola*	Haida Gwaii, BC	Anti-proliferative (a), immuno-stimulatory (b), anti-inflammatory (c) [23]	80% ethanol (a,c) and water (b)	(++), 1 mg/mL (b) (++), 1 mg/mL (c) (+++)
*Laetiporus sulphureus*	Oxford, Ohio	Antimicrobial [26]	Water	MIC = 0.1 mg/mL (+++) against S. epidermidis
*Lentinellus subaustralis*	Oxford, Ohio	Antimicrobial [26]	Water	Against P. aeruginosa, and B. subtilis (+), P. fluorescens, S. epidermidis (MIC = 10 mg/mL), and M. luteus
Lentinus edodes	Quebec	Anti-inflammatory [31]	CDP-like polysaccharide	50 μg/mL (++)
Leucogaster rubescens	Pend Oreille County, Oregon	Antioxidant [25]	95% ethanol	IC_50_ = 20–50 μg/mL (++)
*Leucocybe connata*	Prince George, BC	Anti-proliferative (a), anti-inflammatory (b) [24]	5% NaOH (a) and 80% ethanol (b)	1 mg/mL (a) (++), 1 mg/mL (b) (+++)
*Leucocybe connata*	Prince George, BC	Immuno-stimulatory [24]	Water, 5% NaOH	1 mg/mL (++)
*Leucopaxillus albissimus*	USA	Antimicrobial [36]	2-Aminoquinoline	MIC = 8–65 μg/mL against multidrug resistant clinical isolates (++), 128 μg/mL against A. baumannio (+)
Marasmius oreades	Quebec	Anti-inflammatory [31]	CDP-like polysaccharide	50 μg/mL (++)
Melanogaster tuberiformis	Lane County, Oregon	Antituberculosis (a), anti-inflammatory (b), antioxidant (c) [25]	95% ethanol	IC_50_ = < 20 μg/mL (a) (+++), 50 μg/mL (b) (++), IC50 = > 50 μg/mL (c) (+)
*Paxillus involutus*	Prince George, BC	Anti-proliferative [37]	GIPinv Polysaccharide	IC_50_ = 0.05 mg/mL (+++) on HeLa,0.04 mg/mL (++) on MCF-7
*Phellinopsis conchata*	Oxford, Ohio	Antimicrobial [26]	Water	MIC = 1 mg/mL against S. epidermidis (++)
*Phellinus conchatus*	Oxford, Ohio	Antimicrobial [26]	Water	MIC = 100 mg/mL against S. epidermidis (+++)
*Phellinus igniarius*	Terrace, BC	Anti-proliferative [24]	80% ethanol (a), water (b)	0.2 mg/mL (a) (+++), 1 mg/mL (b) (+)
*Phellinus igniarius*	Terrace, BC	Anti-inflammatory [24]	80% ethanol	1 mg/mL (+++)
*Phellinus nigricans*	Terrace, BC	Anti-proliferative [24]	80% ethanol (a), water (b)	1 mg/mL (a) (+), 1 mg/mL (b) (+)
*Phellinus nigricans*	Terrace, BC	Immuno-stimulatory (a,b), anti-inflammatory (c) [24]	Water (a), 5% NaOH (b), and 50% methanol (c)	1 mg/mL (a) (+), 1 mg/mL (b) (+++), 1 mg/mL (c) (+++)
*Phellodon atratus*	Haida Gwaii, BC	Anti-proliferative, immuno-stimulatory (b & c) [23]	80% ethanol (a), 50% methanol (b) and water (c)	(a) (+), 1 mg/mL (b,c) (+)
*Phellodon atratus*	Haida Gwaii, BC	Anti-inflammatory [23]	80% ethanol (a) and 5% NaOH (b)	1 mg/mL (a) (+++), 1 mg/mL (b) (++)
*Pholiota terrestris*	Oxford, Ohio	Antimicrobial [26]	Water	Against S. epidermidis, P. fluorescens, and M. luteus (++)
*Piptoporus betulinus*	Prince George, BC	Anti-proliferative [24]	80% ethanol (a) and water (b)	0.1 mg/mL (a) (+++), 0.2 mg/mL (b) (+)
*Piptoporus betulinus*	Prince George, BC	Anti-inflammatory [24]	80% ethanol (a), 50% methanol (b) and water (c)	1 mg/mL (a–c)
Pleurotus djamor	Study from Mexico	Anthelmintic activity [38]	Hydro-alcoholic extracts, Pentadecanoic, hexadecanoic, octadecadienoic, octadecanoic acid	40 mg/mL
*Pleurotus levis*	Mexico	Antimicrobial (a) (+++), antioxidant (b) (+++) [39]	Water and alcohol	MIC = 3.33 μg/mL against B. subtilis, 13.32 μg/mL against S. agalactiae (a), 26.64 μg/mL against S. aureus, EC-50 = 0.52 μg/mL (b) (DPPH assay)
Pleurotus ostreatus	USA & Haida Gwaii, BC	Anti-proliferative (a), anti-inflammatory (b) [23]	80% ethanol, 50% methanol	(a) (++), 1 mg/mL (b) (+++)
Pleurotus ostreatus	USA & Haida Gwaii, BC	Immuno-stimulatory [23]	Water	1 mg/mL (++)
Pleurotus ostreatus	USA & Haida Gwaii, BC	Antioxidant (a), Antimicrobial (b) [39]	Water and alcohol	EC_50_ = 1.05 μg/mL (a) (++) (DPPH assay), MIC = 7.83 μg/mL (b) (+) against S. agalactiae
Pleurotus tuber-regium	Olympia, WA	Anti-proliferative (a) [40], antimicrobial (b) [39]	Polysaccharide (a), and water and alcohol (b)	MIC = 6.03 μg/mL (b) (++) against S. agalactiae
Polyporus badius	Oxford, Ohio	Antimicrobial [26]	Water	(+)
Polyporus squamosus	Oxford, Ohio	Antimicrobial [26]	Water	(MIC = 10 mg/mL (isolate 1&2), 100 mg/mL (isolate 3), and M. luteus
Pseudoinonotus dryadeus	Oxford, Ohio	Antimicrobial [26]	100% methanol	(++)
Pyrofomes demidoffi	Oxford, Ohio	Antimicrobial [26]	Water	MIC = 1 mg/mL against S. epidermidis (+++)
Ramaria cystidiophora	Haida Gwaii, BC	Anti-proliferative [23]	80% ethanol (a), 50% methanol (b)	0.1 mg/mL (a) (+++), 0.8 mg/mL (b) (++)
Ramaria cystidiophora	Haida Gwaii, BC	Anti-inflammatory [23]	80% ethanol (a), 50% methanol (b), water (c) and 5% NaOH (d)	1 mg/mL (a-d) (+++)
Ramaria cystidiophora	Vancouver, BC	Antimicrobial [26]	Ramariolide A (22)	MIC = 8 μg/mL against M. smegmatis, 64−128 μg/mL against M. tuberculosis
Rhizopogon couchii	Lebanon State Forest, New Jersey	Anti-inflammatory, antioxidant, antituberculosis [25]	95% ethanol	50 g/mL (a) (++), IC50 = 20–50 μg/mL (b) (++), IC50 = 20–50 μg/mL (b) (++)
Rhizopogon nigrescens	Lebanon State Forest, New Jersey	Anti-inflammatory (a), antioxidant (b) [25]	95% ethanol	50 g/mL (a) (+++), IC50 = > 50 μg/mL (b) (+)
Rhizopogon pedicellus	Pend Oreille County, Oregon	Antioxidant (a), antituberculosis (b) [25]	95% ethanol (a),70% ethanol (b)	IC_50_ = 20–50 μg/mL (a) (++), IC_50_ ≥ 50 μg/mL (b) (+)
Rhizopogon subareolatus	Lewis County, Washington	Antimalarial [25]	95% ethanol (+)	15.9 μg/mL (+)
Rhizopogon subaustralis	Lebanon State Forest, New Jersey	Anti-inflammatory (a), antioxidant (b) [25]	95% ethanol	IC_50_ ≥ 50 μg/mL (a) (+), 50 μg/mL (b) (+++)
Rhizopogon subgelatinosus	Jackson County, Oregon	Anti-inflammatory (a), anti-proliferative [25]	95% ethanol	50 μg/mL (a)
Russula paludosa	Haida Gwaii, BC	Anti-proliferative [23]	80% ethanol (a), 50% methanol (b), water (c), and 5% NaOH (d)	(a–d) (+)
Russula paludosa	Haida Gwaii, BC	Anti-inflammatory [23]	80% ethanol (a), 50% methanol (b), water (c)	1 mg/mL (a–c) (+++)
Scleroderma laeve	Lebanon State Forest, New Jersey	Anti-inflammatory (a), antioxidant(b), antituberculosis (c) [25]	95% ethanol (+++)	50 μg/mL (a), IC50 = < 20 μg/mL (b), IC50 = < 20 μg/mL (c)
Stereum hirsutum	Oxford, Ohio	Antimicrobial [26]	Water	Against B. subtilis, S. epidermidis, and P. fluorescens (+)
Trametes versicolor	Oxford, Ohio	Antimicrobial [26]	Water	MIC = 10 mg/mL against S. epidermidis (+++)
*Trichaptum abietinum*	Prince George, BC	Anti-proliferative [24]	80% ethanol (a) and water (b)	0.2 mg/mL (a) (++), 0.2 mg/mL (b) (+)
*Trichaptum abietinum*	Prince George, BC	Immuno-stimulatory (a), anti-inflammatory (b) [24]	50% methanol (a) and 5% NaOH (b)	1 mg/mL (a) (+++), 1 mg/mL (b)
*Tricholomopsis rutilans*	Haida Gwaii, BC	Anti-proliferative [23]	80% ethanol	(++)
*Tricholomopsis rutilans*	Haida Gwaii, BC	Anti-inflammatory [23]	80% ethanol (a), 50% methanol (b) and water (c)	1 mg/mL (a–c) (+++)
*Tyromyces chioneus*	Haida Gwaii, BC	Anti-proliferative (a), immuno-stimulatory (b) [23]	80% ethanol (a) and water (b)	(a) (+), 1 mg/mL (b) (++)
*Tyromyces chioneus*	Haida Gwaii, BC	Anti-inflammatory [23]	80% ethanol (a) and m50% ethanol (b)	1 mg/mL (a) (+++), 1 mg/mL (b) (++)
*Urnula craterium*	La Crosse, Wisconsin	Antimicrobial [41]	Urnucratin A (27) Urnucratin B (28) (++) Urnucratin C (29) (+++)	(27) MIC = 2 μg/mL (+) (methicillin-resistant S aureus), 1 μg/mL (vancomycin-resistant E. faecium), 0.5 μg/mL (S. pyogenes)

+++ refers to strong activity, ++ moderate activity, + weak activity. ^1^ Refer to Table 2 for more information of bioactive small molecules and polysaccharides. ^2^ All studies were conducted in vitro, except where indicated. (a), (b), (c) and (d) in Column 4 (Bioactive Component or Extraction Solvent) are referred to in Column 5 (Active Dose).

**Table 2:** Bioactive Molecules from Mushrooms Native to North America.

Types	Bioactive Compound	References	Mushrooms
Small molecules	Syringaldehyde (1)	[29]	*E. granulatus*
	Syringic acid (2)	[29]	*E. granulatus*
	Grifolin (3)	[21,22]	*A. flettii*
	Neogrifolin (4)	[21,22]	*A. flettii*
	Confluentin (5)	[21,22]	*A. flettii*
	3,11-Dioxolanosta-8,24(*Z*)-diene-26-oic acid ^1^ (6)	[21]	*J. hirtus*
	Erinacerin V ^1^ (7)	[17]	*Hericium* sp.
	4-Hydroxy-2,2-dimethylchromane-6-carbaldehyde ^1^ (8)	[17]	*Hericium* sp.
	4-Chloro-3,5-dimethoxybenzaldehyde (9)	[17]	*Hericium* sp.
	2-Chloro-1,3-dimethoxy-5-methyl benzene (10)	[17]	*Hericium* sp.
	4-Chloro-3,5-dimethoxyphenylmethanol (11)	[17]	*Hericium* sp.
	3,6-Bis(hydroxyl methyl)-2-methyl-4*H*-pyran-4-one (12)	[17]	*Hericium* sp.
	4-Chloro-3,5-dimethoxybenzoic acid (13)	[17]	*Hericium* sp.
	5-Hydroxy-6-(1-hydroxyethyl)isobenzofuran-1(3*H*)-one (14)		*Hericium* sp.
	Erinacine (15)	[17]	*Hericium* sp.
	Pentadecanoic acid (16), Hexadecanoic acid (17), Octadecadienoic acid (18), Octadecanoic acid (19)	[38]	*P. djamar*
	Orellanine (3,3′,4,4′-tetrahydroxy-2,2′-bipyridine-1,1′-dioxide) (20)	[19]	*C. armillatus*
	Ramariolide A ^1^ (21)	[50]	*R. cystidiophora*
	Ramariolide B ^1^ (22)	[50]	*R. cystidiophora*
	Ramariolide C ^1^ (23)	[50]	*R. cystidiophora*
	Ramariolide D ^1^ (24)	[50]	*R. cystidiophora*
	2-Aminoquinoline (25)	[36]	*L. albissimus*
	Urnucratin A ^1^ (26)	[41]	*U. craterium*
	Urnucratin B ^1^ (27)	[41]	*U. craterium*
	Urnucratin C ^1^ (28)	[41]	*U. craterium*
	3-Oxo-24-methyl-5α-lanost-8,25-dien-21-oic acid ^1^ (29)	[30]	*F. pinicola*
	Pinicolic acid (30)	[30]	*F. pinicola*
	Polyporenic acid (31)	[30]	*F. pinicola*
	16α-Hydroxy-24-methylene-3-oxo-5α-lanost-8-ene-21-oic acid (32)	[30]	*F. pinicola*
	16α-Acetyloxy-24-methylene-3-oxo-5α-lanost-7,9(11)-dien-21-oic acid (33)	[30]	*F. pinicola*
	22E-5α-Ergost-7,9(11),22-trien-3β-ol (34)	[30]	*F. pinicola*
Large molecules	GIPinv ^1^	[37]	*P. involutus*
	CDP ^1^	[31,32]	*G. dryophilus*
	AlPetinc ^1^	[18]	*E. tinctorium*
			
	CDP-like polysaccharide ^1^	[31]	*L. edodes*
	CDP-like polysaccharide ^1^	[31]	*M. oreades*
	EPS ^1^	[52]	*P. tuber-regium*
			
	Y3 ^1^	[28]	*C. comatus*

^1^ New compounds.
